# Complementary serum proteomic analysis of autoimmune hepatitis in mice and patients

**DOI:** 10.1186/1479-5876-11-146

**Published:** 2013-06-13

**Authors:** Hongbin Li, Guoshun Li, Xinyu Zhao, Yongkang Wu, Wen Ma, Yuling Liu, Fengming Gong, Shufang Liang

**Affiliations:** 1 State Key Laboratory of Biotherapy and Cancer Center, West China Hospital, Sichuan University, Chengdu 610041, P. R. China; 2 West China Hospital, West China Medical School, Sichuan University, Chengdu 610041, P. R. China

**Keywords:** Autoimmune hepatitis, Serum proteomics, Isobaric tags for relative and absolute quantification (iTRAQ)

## Abstract

**Background:**

Autoimmune hepatitis (AIH) is a chronic liver disease caused by inflammation of the liver. The etiology of AIH remains elusive, and there are no reliable serum biomarkers.

**Methods:**

In order to identify candidate biomarkers, 2-DE analysis of serum proteins was performed using a mouse model of AIH induced by treatment with concanavalin A (ConA). To enrich samples for low abundance molecules a commercial albumin removal reagent was used. In an independent analysis, candidate biomarkers were identified in AIH patient’s serum by a targeted iTRAQ (isobaric tags for relative and absolute quantification) identification. Candidates were validated in independent cohorts of ConA treated mice and AIH patients by ELISA (enzyme-linked immuno sorbent assay).

**Results:**

Nine proteins were differentially expressed in AIH mice treated with con-A. Two of these, the third component of complement (C3) and alpha-2-macroglobulin (A2M) were also up-regulated in AIH patient’s sera by a targeted iTRAQ identification. In separate validation studies, serum C3 and A2M levels were increased in mice with ConA treatment after 20-40 h and in 34 AIH patients in a subgroup analysis, females with AIH aged 20–50 years old displayed the largest increases in serum A2M level. Biological network analysis implements the complement cascade and protease inhibitors in the pathogenesis of AIH.

**Conclusion:**

The serum proteins C3 and A2M are increased both in a mouse model and in patients with AIH by both 2-DE and iTRAQ methods. This integrated serum proteomics investigation should be applicable for translational researchers to study other medical conditions.

## Background

Autoimmune hepatitis (AIH) is a chronic liver-specific disease, characterized by elevated aminotransferase levels, autoantibodies, increased γ-globulin or IgG levels and biopsy evidence of interface hepatitis [1,2]. The etiology of AIH is not completely understood at present, which makes it difficult to diagnose [3]. The clinical diagnosis still depends on the exclusion of other causes of chronic hepatitis and use of an international scoring system that includes epidemiologic, biochemical, histological and serologic criteria [4-6].

In clinical examinations, these autoantibodies, including anti-nuclear antibodies, anti-smooth muscle antibodies, antibodies to liver-kidney-microsome type 1 and antibodies to liver cytosol type 1, are used to distinguish subtypes of AIH. However these antibodies are not specific to AIH as they are also found in sera from patients with viral hepatitis, drug-induced hepatitis, or other autoimmune diseases [7]. Therefore, special biomarkers for the diagnosis of AIH are still urgently needed.

In the past years, several groups have applied various approaches to look for potential markers for AIH. Scientists have found some candidate biomarkers for AIH by proteomics tools. For example, a heterogenous nuclear ribonucleoprotein A2/B1 (hnRNP-A2/B1) has been identified as an autoantigen in type 1 AIH (AIH-1) by immune proteomics for the first time [8]. Similarly, fumarate hydratase and phosphoglycerate mutase isozyme B are also identified as candidate autoantigens in Chinese patients with AIH [3,9]. Tahiri et al. demonstrated liver arginase, HSP60, HSP70, HSP90 and valosin-containing protein represent potential targets on liver membrane for autoantibodies in AIH-1 by immunofluorescence microscopy [10]. In a recent study, the chip technology was widely used in identification of biomarkers [6,11]. Six autoantigens (IL4R, AL137145, LOC646100, C17orf99, METRNL, APCDD1L) were specifically recognized by AIH sera [11]. In addition, serological biomarkers were screened from recombinant cDNA expression libraries. And several molecules, such as lamin, histone, cyclin A and U1RNP-A, have been identified as autoantigens in AIH [12].

However, the discrepancy and non-specificity in previous reports are obvious for these candidate antigens, which are probably due to the limitations in sensitivity and accuracy for different approaches. For example, heat shock proteins (HSPs), differentially expressed in AIH [13,14] are usually overexpressed in many human cancers and are implicated in tumor cell proliferation, differentiation, invasion and metastasis [13,14]. Especially, several HSPs, including HSP27 and HSP70 etc., are implicated with the prognosis of specific cancers. In addition, the individual differences of AIH patients also result in diverse identifications of potential biomarkers.

In order to overcome discrepancy of different analytical methods, as well as understand the pathogenesis of AIH, we adopted a comprehensive serum-based proteomics strategy to look for serological molecules, especially relative to autoimmune characterization in AIH. The application of proteomics to discovery of serum biomarkers is very active and promising for AIH at present. However, serum proteomics presents tremendous technical challenges, with its vast dynamic range in protein concentrations spanning at least ten of orders of magnitude and their dominance by a relatively small number of highly abundant proteins with about 20 proteins constituting 99% of the entire protein content of serum [15]. So far it is challenging to look for low abundance biomarkers by serum proteomics analysis. Our studies include a comparative serum proteome profiling on AIH mouse models and a targeted iTRAQ (isobaric tags for relative and absolute quantification) quantitative proteomic validation for sera from AIH patients, and subsequently complement C3 (C3) and alpha-2-macroglobulin (A2M) are both identified to associate with AIH in mouse model and patient’s serum. The potential serological molecules will provide new viewpoints for understanding AIH. This integrated serum proteomics investigation based on AIH animal models and patient’s sera can overcome discrepancy in samples and analytical tools, which provides a complementary means to look for potential AIH biomarkers.

## Results

### Mouse model with autoimmune hepatitis

**Figure 1:**
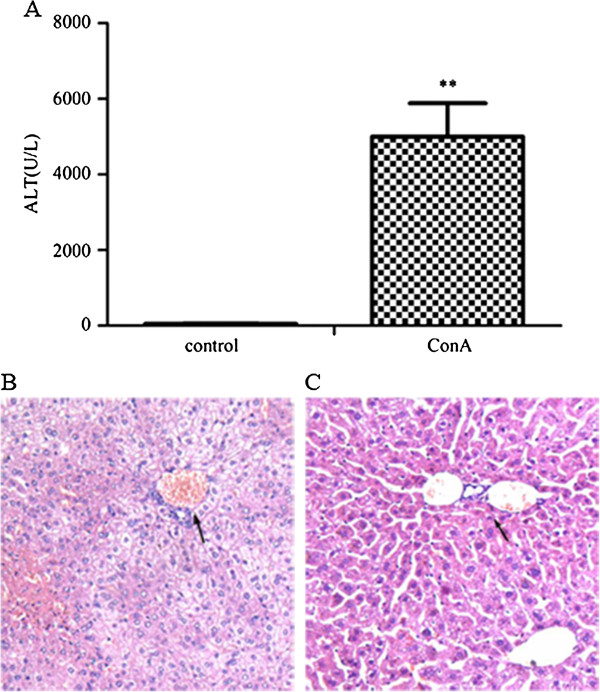
**The characterization of a mouse model with autoimmune hepatitis.** The serum ALT levels **(A)** and histological examination of liver tissues **(B-C)** were shown. The ALT levels were elevated in serum at 20 h after ConA treatment for BALB/c mice (n = 6, **p < 0.01). The hepatic vein showed pathological changes obviously (arrow indications) in liver biopsy of AIH mice **(B)**, while the hepatic portal vein and hepatic lobule are clear in normal mouse liver **(C)**.

We first successfully developed a mouse model with AIH by concanavalin A (ConA) treatment. The pathological evidences demonstrated that this model had characterization of AIH [16,17]. In addition, abnormally high levels of alanine transarninase (ALT) also showed that hepatocytes had been damaged. In the AIH mouse model, the serum levels of ALT in mouse were elevated significantly after ConA injection for 20 h (n = 6, p < 0.01) (Figure 1A), and livers from ConA-induced hepatitis mice exhibited inflammatory cell infiltration, congestion and dilatation of blood vessel, as well as widespread hepatocellular necrosis in liver lobules (Figure 1B/C). Also pathological changes of hepatic portal vein had been observed on tissue sections obviously.

### Removal efficiency of serum high-abundant proteins

Due to the extensive source, rich value of application, serum proteomics has important clinical applications on screening diagnostic markers. However, serum albumin can constitute about 50-70% of the total serum proteins and IgG is up to 10-25%. It is the high abundance of serum albumin and IgG that are main drawback of a comprehensive analysis of body fluids. The presence of these high-abundant proteins can mask some low-abundant proteins and cause loss of resolution in 2-DE [18].

**Figure 2:**
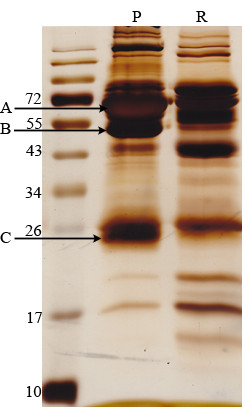
**Removal efficiency of serum high-abundant proteins.** 40 μl of healthy human plasma was processed with the ProteoExtractTM Albumin Remove reagent. 50 μg protein from each fraction was separated by SDS-PAGE and visualized by silver staining. The aggregation of HAS **(A)**; IgG heavy chain **(B)** and IgG light chain **(C)** was greatly reduced. P: human plasma, R: plasma without high-abundant proteins.

After depletion of serum high-abundant proteins by using the ProteoExtract™ Albumin/IgG Remove Kit, more than 80% of serum albumin was removed from serum as monitored by analysis of stained bands on SDS-PAGE (Figure 2). And the aggregation of human serum albumin (HAS), IgG heavy chain and IgG light chain was greatly reduced. The AIH mouse sera with depletion of high-abundant proteins were run 2-DE, and the treated sera from AIH patients were performed iTRAQ analysis.

### Differential serum expressing profiling in 2-DE gel

**Figure 3:**
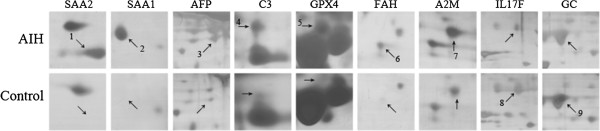
**The spots with differential expression level between AIH and normal mouse sera.** Spots labeled with the arrow were significantly changed. The spot 1–7 was up-regulated in AIH mouse serum, while the spot 8–9 was significantly decreased. The protein corresponding to the spot 1–9 was respectively identified as following, including SAA2, SAA1, AFP, C3, GPX4, FAH, A2M, IL17F and GC.

The differential serum expression profile between AIH mice and controls was compared by 2-DE. Several differently expressed proteins were enlarged to show in Figure 3. The experiments were repeated three times independently. Only reproducibly detected spots with statistical significance were subjected to mass spectrum (MS) analysis. A total of 9 proteins displayed marked alterations and were successfully identified by MS/MS between AIH and control sera (±over 1.5-fold, p < 0.05) (Table 1).

**Table 1:** Differentially expressed proteins identified by 2-DE

**Spot no.**	**Protein**	**Gene name**	**Function**	**Accession**	**Theoretical M**_**r**_**/pI**^**a**^	**Score**^**b**^	**No. of pep.**^**c**^	**Expression level (AIH/Control) **^**d**^	**iTRAQ validation (116/114)**^**e**^
1	serum amyloid A2	SAA2	acute phase reactant	gi6755394	13613.5/6.41	122	2	↑6.56 ± 0.2	N/A^f^
2	serum amyloid A1	SAA1	acute phase reactant	gi404753	13691.5/6.5	45	2	↑14.72 ± 2.1	N/A
3	alpha-fetoprotein	AFP	estrogens, fatty acids and metals binding	gi191765	47194.9/5.47	172	7	↑3.00 ± 0.9	N/A
4	complement C3	C3	activation immune	gi126518317	187904/6.31	44	3	↑2.41 ± 0.06	2.19
5	glutathione peroxidase	GPX4	oxidative damage protecting calcium ion binding	gi2673845	22290.59/6.74	54	3	↑4.16 ± 0.18	N/A
6	fumarylacetoacetase	FAH	enzyme regulator activity; protein binding	gi253320	46416/6.56	43	4	↑12.4 ± 0.78	N/A
7	alpha-2-macroglobulin	A2M	activation immune	gi110347469	167144/6.17	41	3	↑1.98 ± 0.23	1.61
8	interleukin 17 F	IL17F	actin/ vitamin D binding	gi22003916	17008.8/9.05	40	2	↓8.3 ± 1.11	N/A
9	vitaminD-binding protein precursor	GC	vitamin transporter activity	gi51172612	53565/5.39	56	3	↓2.08 ± 0.2	N/A

^a^Theoretical molecular weight (kDa) and pI from the ExPASy database.

^b^Probability-based Mascot scores.

^c^Number of unique peptides identified by MS/MS.

^d^Average expression level of altered proteins by 2-DE and MS from three independent analyses (mean ± SD) (↑,increased; ↓, decreased).

^e^The differential expression of proteins in serum from AIH patients and controls was validated by iTRAQ (average of the two experiments, control: 114; AIH: 116).

^f^N/A, Not analyzed by iTRAQ.

Among the 9 differentially expressed proteins, the average number of unique peptides identified by MS/MS was 3.2, and the average value of Mascot score was 66. Among them, interleukin 17 F and vitamin D-binding protein precursor were down-regulated in AIH, while other 7 proteins were up-regulated. Most altered proteins belong to immune or hepatitis related proteins. In the increased proteins, serum amyloid A1 (SAA-1) and serum amyloid A2 (SAA-2) are acute phase lipoproteins rapidly produced in the liver with trauma or infection, and the serum amyloid proteins potently enhance cytokine production from peripheral blood mononuclear cells. In our data the SAA-1 and SAA-2 were indentified to increase with the highest degree, which is also supported that SAA has a high correlation with hepatitis [19]. Prior studies have shown that the serum concentrations of alpha-fetoprotein, variably elevated during liver injury, have been suggested to be of prognostic importance in acute liver failure [20]. Similarly, Kuo et al. found a marked elevation of serum alpha-fetoprotein with severe jaundice as an initial manifestation of AIH [21]. The fumarylacetoacetase, which was also identified to be upregulated in our AIH mice, was also verified by its autoantibody in sera of a mouse hepatitis model [22]. The findings brought into correspondence with our experimental result which showed an increase in AIH model compared to the control group.

However, we paid attention to two up-regulated proteins C3 and A2M, tightly associated with immune response in autoimmune inflammation. Although these two proteins were not altered with a largest degree, in order to validate their relative expression levels in patient’s serum with AIH, a targeted iTRAQ analysis as a proof-of-principle experiment was performed to examine the expression of C3 and A2M in AIH patient’s serum as following.

### The iTRAQ-based validation for two proteins in patient’s serum

**Figure 4:**
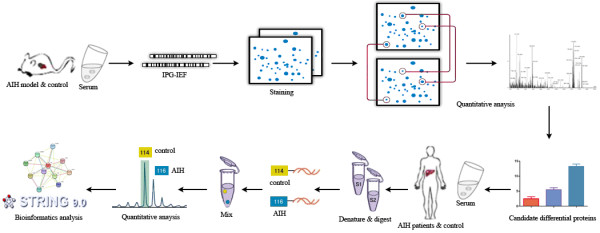
**The serum proteome analysis of a mouse model with autoimmune hepatitis by 2-DE and a targeted iTRAQ validation on clinical serum.** The pooled serum samples were depleted of albumin and IgG using ProteoExtract Albumin Removal Kit, and protein fractions were used for the subsequent differential expression analysis. Serum protein expression profile of AIH mice was compared with normal mouse serum by 2-DE, differential proteins were identified by MS/MS. On the other hand, the AIH patient’s serum was pretreated and labeled with iTRAQ reagents to perform LC-MS/MS, and the target proteins were selected to validate their expression levels in AIH patient’s sera by iTRAQ quantification.

As a proof-of-concept investigation, the altered serum proteins C3 and A2M in AIH mouse were further verified their expression levels in AIH patient’s serum by a targeted iTRAQ method. The strategy of this process was elucidated in Figure 4. The differential expression level between normal and AIH serum was determined *via* the peak intensity ratio of iTRAQ-labeling peptides with a mass tag (m/z116, m/z114).

**Figure 5:**
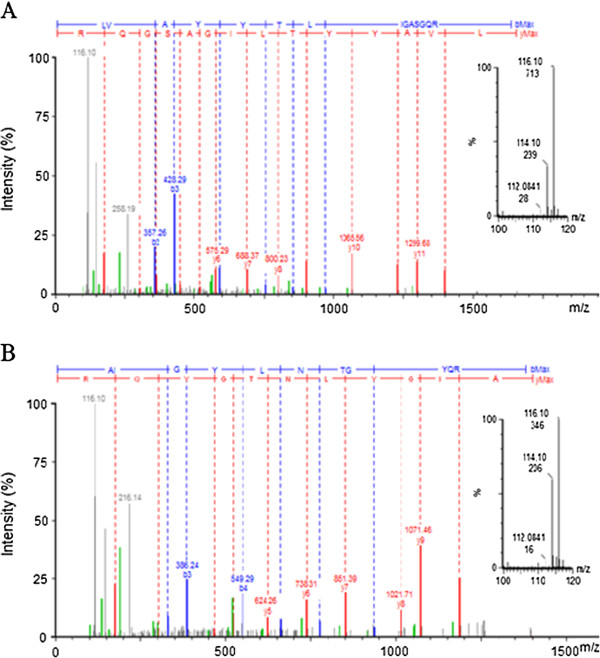
**Representative MS/MS spectra of iTRAQ-labeling peptides derived from C3 and A2M.** For each MS/MS spectrum, b- and y-type fragment ions enable peptide identification, whereas the intensity counts of peak areas for each of the iTRAQ signature ions enable quantification of the peptides and proteins. **A**, MS/MS spectra of the peptide “LVAYYTLIGASGQR” from complement C3β. **B**, MS/MS spectra of the peptide “AIGYLNTGYQR” from A2M. The pair of reporter ions (m/z116 and m/z114) was highlighted in the enlarged inset map.

Consistent with 2-DE results in AIH mouse, the C3 and A2M were also identified to increase in patient’s serum with AIH by iTRAQ analysis (Figure 5). The peptides identified by iTRAQ were listed in the Table 2. For example, the peak intensity of a peptide containing “LVAYYTLIGASGQR” sequences in protein C3 was 713 and 239 counts corresponding to the 116 and 114 reagent labeling serum (AIH serum *versus* normal serum). Therefore, the differential expression level of C3 was 2.98-fold upregulation in AIH patient’s serum compared with the normal human serum in first time iTRAQ analysis. Among these isotope labeling peptides of C3, the isotope peptides “^531^LVAYYTLIGASGQR^544^” was specifically derived from C3 β chain, one of breakdown products of C3. And this pair of peak intensity ratio was 2.9 (116 versus 114), which mainly contributed to the increased level of C3 in serum. Further, the antibody for ELISA analysis was raised against peptides mapping near the N-terminus of human C3, allowing for detection of the beta-chain in both the uncleaved C3 precursor protein and a cleaved product C3b. Similarly, the 116/114 mass containing peptide intensity ratio was 1.68 (346/206 = 1.68) in the A2M, which indicated that it was increased in AIH serum. These two kinds of methods showed a high degree of consistency.

**Table 2:** The peptides of C3 and A2M were identified by iTRAQ analysis

**Protein name**	**Peptide sequence**	**Ion score**^**a**^	**Peptide ratio (116/114)**^**b**^	**Protein ratio (116/114)**^**c**^
alpha-2-macroglobulin	AIGYLNTGYQR.	43	1.708	1.61 ± 0.14
	GVPIPNK.	19	1.496	
	SASNMAIVDVK.	75	1.727	
	SIYKPGQTVK.	48	1.401	
	ALLAYAFALAGNQDKR.	77	1.499	
complement C3^d^	LVAYYTLIGASGQR	77	2.9	2.19 ± 0.78
	AGDFLEANYMNLQR	48	1.363	
	YFKPGMPFDLMVFVTNPDGSPAYR	45	1.385	
	DQLTCNK	42	1.316	
	TGLQEVEVK	54	1.932	

^a^Probability-based Mascot scores.

^b^The peptides of C3 and A2M were identified by iTRAQ analysis (Control: 114; AIH: 116).

^c^The differential expression of C3 and A2M in serum between AIH patients and controls was calculated based on the isotope labeling peptides.

^d^ Peptide “^531^LVAYYTLIGASGQR^544^” is a part of C3β chain.

Peptide “^1172^AGDFLEANYMNLQR^1185^” is a part of C3d fragment.

Peptide “^363^YFKPGMPFDLMVFVTNPDGSPAYR^386^” is a part of C3β chain.

Peptide “^1354^DQLTCNK^1360^” is a part of C3c α chain fragment 2.

Peptide “^905^TGLQEVEVK^913^” is a part of C3c α chain fragment 1.

### Serum levels of C3 and A2M in AIH mice measured by ELISA

**Figure 6:**
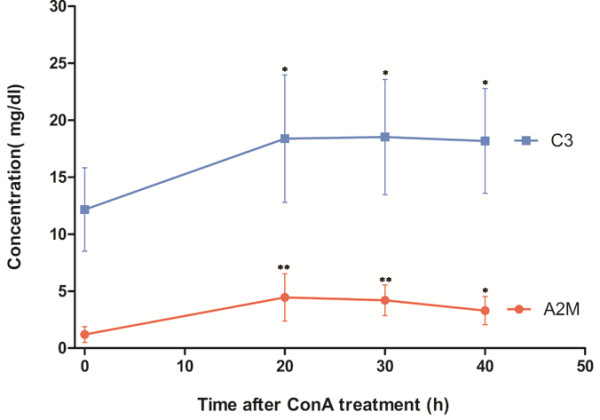
**Serum Levels of C3 and A2M in AIH mice with ConA treatment for different times.** In each group, six mice were treated with ConA from 0–40 h (n = 6). *P <0.05, **P < 0.01 (AIH mouse serum *versus* control serum). Data were shown with the mean value and SD.

In order to validate different levels of C3 and A2M in normal conditions and pathological disease with AIH, their serum levels were dynamically detected in AIH model mice. Female BALB/C mice, 6–8 weeks old, were used to establish animal models with AIH. Mice serum samples were collected at different time points after ConA treatment (20 h, 30 h, 40 h) for ELISA analysis. At each treatment time point, 6 mice were used as a group to detect serum protein level compared with a standard curve, and each serum sample was detected in triplicate. In the control group without ConA treatment, serum C3 and A2M was in a lower level, with 12.16 ± 3.67 mg/dl for C3 and 1.19 ± 0.69 mg/dl for A2M. While serum level of C3 and A2M was ascended sharply to 19.79 ± 6.13 mg/dl and 4.45 ± 1.8 mg/dl after ConA treatment for 20 h, and their serum concentration was not changed significantly in the next two treatment time points of 30 h, 40 h (Figure 6).

### ELISA examination on serum levels of C3 and A2M in AIH patients

First, we focused on the general serum level of these two proteins in 34 AIH patients, which was consistent with the higher level in AIH mouse models. In 34 AIH patients, the average value of serum C3 was 210.72 ± 51.43 mg/dl, while the mean value of normal controls was 156.27 ± 41.83 mg/dl. So the serum C3 was increased in AIH patients compared with normal persons (p<0.01). Among the 34 AIH cases, serum C3 level was increased in 25 AIH samples (73.5%) compared to the normal controls, and 5 samples (15%) were no obvious differences between AIH and control group (Table 3). However, C3 level was lower in other 4 cases (11.8%) of AIH serum than normal human donors. Similarly, the average value of serum A2M in AIH patients was 161.45 ± 91.49 mg/dl, which was higher than the average level 102.94 ± 44.03 mg/dl in normal persons (p<0.01). Among the 34 AIH samples, serum A2M level was increased in 21 AIH patients (61.8%), 4 cases (11.8%) had similar levels between AIH and control group. There were also 9 cases (26.4%) of AIH with lower serum A2M level than normal persons.

**Table 3:** The serum level of C3 and A2M were compared between AIH and control group

	**Relative serum level**^**a**^		
	**Increased**	**Little changed**	**Decreased**
**Protein name**	**Sample cases (%) **^**b**^		
C3	25 (73.5%)	5 (14.7%)	4 (11.8%)
A2M	21 (61.8%)	4 (11.8%)	9 (26.4%)

^a^ Compared with the control group, the level of the protein in AIH group.

^b^ Sample cases and the percentage of the cases compared with the total.

**Figure 7:**
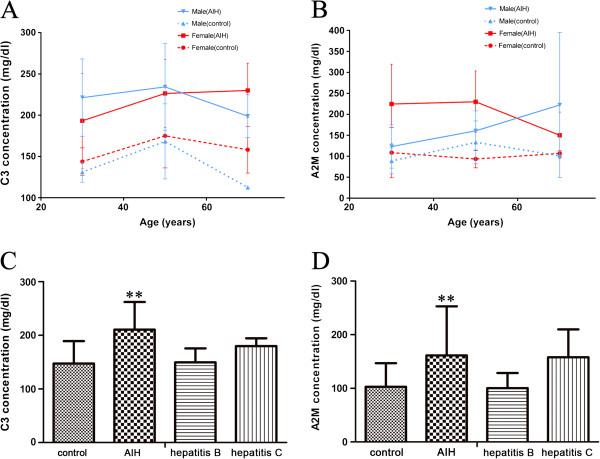
**Serum C3 and A2M levels in AIH patients validated by ELISA. A-B**. Serum C3 and A2M levels measured based on the gender and age of AIH patients . (Classified by gender, n = 10 for female control group and n = 16 for female AIH group; n = 9 for male control group and n = 18 for male AIH group. Classified by age in AIH group, n = 13 for patients with 20–40 years, n = 13 for the group with 41–60 years, and n = 8 for the group with 61–80 years. For control healthy group, n = 7 for 20–40 years’ group, n = 6 for 41–60 years’ group and n = 5 for 61–80 years’ group). **C-D**. Serum C3 and A2M levels measured in patients with different forms of hepatitis. (**P < 0.01 *versus* control samples, n = 10 for hepatitis B group, n = 10 for hepatitis C group and n = 19 for control group).

Subsequently, we classified serum samples according to patient’s gender and age (Additional file 1: Table S1), and serum C3 and A2M levels were further analyzed between male and female patients in different age groups. As shown in Figure 7A, there were markedly elevated levels of serum C3 and A2M in AIH persons than healthy donors in the age range of 20–70 years. It was noted that serum A2M level in female AIH patients was higher than the male ones from 20–60 years (p<0.01). Particularly, in young and middle-aged AIH persons with 20–50 years, serum A2M level was greatly increased significantly in female group (Figure 7B). Our results were also supported previous conclusions that AIH can be found more often in younger patients with prevalence for females [23].

### Comparisons of C3 and A2M level in different forms of hepatitis

Furthermore, serum levels of these two proteins were measured in other forms of hepatitis, including patients with hepatitis B and hepatitis C, to primarily validate their sensitivity and specificity in AIH. As results, compared with 10 cases with hepatitis B or hepatitis C, the serum C3 and A2M were raised in AIH patients than healthy persons with significant differences (Figure 7C-D), while their levels were remained unconspicuous differences among groups with hepatitis B or hepatitis C, and healthy controls. So far, the serum levels of C3 and A2M in AIH patients can directly reflect immunopathological conditions.

### Biological network analysis by web-based bioinformatics analysis

The C3 and A2M were revealed as differentially expressed between AIH and normal serum validated by 2-DE, iTRAQ and ELISA analysis. In order to analyze potential roles of the two proteins in inflammatory disease, the intrinsic interactions with other proteins of C3 and A2M were respectively analyzed using STRING software, a web-based protein interaction mapping tool.

**Figure 8:**
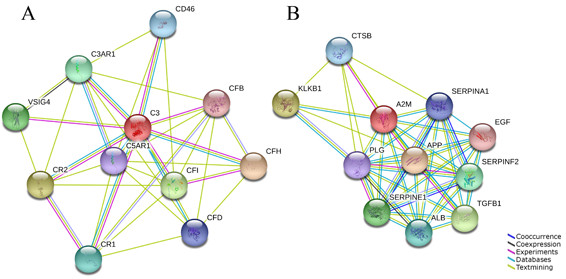
**Proteins involved in complement C3 and A2M functions were analyzed using STRING software.** The functional partners were predicted by different methods, which were shown in different line colors (Seen the panels within the figure caption). Blue (, co-occurrence), means the presence of linked proteins across species; black (, coexpression), means the genes co-expressed in the same or in other species; purple (, experiments), shows a significant protein interaction from the reports of literatures; light blue (, database), shows a significant protein interaction groups gathered from databases; green (, textmining), shows protein interaction groups extracted from scientific literatures. The predicted functional partners include as follows. CFH, complement factor H; CR2, complement component receptor 2; CFI, complement factor I; VSIG4, V-set and immunoglobulin domain containing 4; C3AR1, complement component 3a receptor 1; CR1, complement component (3b/4b) receptor 1; CD46, CD46 molecule, complement regulatory protein; CFD, complement factor D; C5AR1, complement component 5a receptor 1; CFB, complement factor B; APP, Amyloid beta (A4) precursor protein; KLKB1, plasma kallikrein; TGFB1, transforming growth factor, beta 1; SERPINE1, serpin peptidase inhibitor, clade E, member 1; SERPINF2, serpin peptidase inhibitor; ALB, albumin; CTSB, cathepsin B; SERPINA1, serpin peptidase inhibitor, clade A, member 1; PLG, plasminogen; EGF, epidermal growth factor.

In the protein interaction maps, ten proteins including complement factor H, complement component receptor 2 (CR2), complement factor I all interact with C3 (Figure 8A). The complement cascade and its regulation by these other proteins have all been described in great detail. C3 plays a central role in the activation of the complement system by the classical, alternate, and lectin pathways. Factor H functions as a co-factor in the inactivation of C3b by factor I and also increases the rate of dissociation of the C3bBb complexes. CR2 also regulates complement activation, serves as a receptor for iC3b, C3dg, and C3d, and contributes to B lymphocyte activation [24].

Another upregulated protein, A2M also has tightly associated with other 10 proteins in an immune response network (Figure 8B). Amyloid beta (A4) precursor protein and transforming growth factor beta 1 assist A2M to participate in exocytosis of platelet alpha granule contents. And serpin peptidase inhibitors, clade E and clade F, are also involved in this process. Another molecule, plasma kallikrein is significant for the counterbalance of plasma renin angiotensin system which may mediate effects on the immune response in autoimmune inflammation [25,26].

## Discussion

In this study, we had applied a comprehensive serum proteomics strategy to look for important differential proteins in serum based on AIH mouse model and patient’s serum. And totally 9 altered proteins were identified in AIH mice serum by 2-DE. Two upregulated proteins, C3 and A2M, were validated in the serum of AIH patients by a targeted iTRAQ analysis. And furthermore, serum level of C3 and A2M was generally higher in 34 cases of AIH patients than normal persons by ELISA detection. From mouse models to clinical AIH sera, the integrated serum proteomics investigation can overcome discrepancy in samples and tools, which is a translational medical viewpoint to look for the molecules associated with AIH.

Serum proteome contains the proteins not only from the liver, small intestine synthesis such as albumin, but also millions of species of immunoglobulin. The serum proteome holds the promise of a reform in disease diagnosis and therapeutic monitoring provided that major challenges in proteomics and related disciplines can be addressed [15,27]. The iTRAQ-based quantitative proteomics can offer a discovery of more accurate differences of expression between different samples. Based on the initial attempt to analyze the patient’s serum with AIH by iTRAQ method, more potential biomarkers will be found in latter systematically identification on multiple serum samples.

Autoantibodies make a major contribution to the diagnosis of autoimmune diseases, and immunoproteomics provides a wide range of application for AIH research. So there are many studies to look for autoantibody-based biomarkers. On the perspective of specific autoantigens, comprehensive serum proteomics strategy has been used to look for differential proteins in AIH. Beside the potential diagnostic value, the discovery of novel AIH autoantigens could provide insights on immunopathological conditions and pathogenicity mechanism of this disease. In our study, several proteins were identified to increase in AIH serum, which had been reported in other previous studies. For example, it has reported that the elevation of AFP level can be caused by autoimmune hepatitis [21]. And this protein AFP was also identified to increase to 3 times in AIH mouse serum in our analysis. So far, our work could provide some values for identification of specific autoantigens of AIH through a translational medical strategy coupled with an integrated proteomic approach.

On the other hand, the C3 and A2M both belong to inflammatory and immune-relative molecules. C3 is an acute phase reactant of the complement system that represents a crucial effector of the acute phase response of innate immunity. Excessive complement activation and abnormal serum levels of C3 and A2M have been implicated in the pathophysiology of various inflammatory diseases [28-30], including AIH, systemic lupus erythematosus (SLE) [31], diabetes [32], and anorexia nervosa [30], etc. In order to primarily validate the sensitivity and specificity of C3 and A2M in AIH, serum levels of these two proteins were measured in other forms of hepatitis, including 10 patients with hepatitis B or hepatitis C. Therefore, serum levels of C3 and A2M can serve as markers of some disease activity, even a potential biomarker in relation to diagnosis for the disease severity.

In our investigation, the C3 level was identified to increase in AIH serum. Activation of the complement system is involved in the pathogenesis of the systemic autoimmune diseases [29]. C3 plays an important role in the activation of the complement system. The C3 lies in the central position to interact other complement components from our bioinformatics analysis. The up-regulation of C3 and its interacting network suggest that it plays an important role in the development of AIH. Therefore, the change quantification of C3 level in AIH patient’s serum is key to understand the alterations that biological systems undergo. Recent studies have demonstrated that complement contributes to the development of autoimmune diabetes [33,34]. Also reports showed that complement could play a pivotal role in liver specific autoantibody, which mediated hepatocyte injury in AIH, and that complement inhibitors could be, in principle, developed as novel therapeutics against AIH [35].

A2M levels and change patterns in AIH patient’s serum can differ greatly between males and females, although generally its concentration in AIH is significantly higher than normal human serum (Figure 7B). In fact, the serum A2M level is usually different between man and woman in normal physiological conditions [28], which partially brings about more complexity in level changes in immunopathological conditions. Similarly, this difference of A2M level is also associated with age and sex in diabetes [32]. Furthermore, serum A2M level is significantly increased in patients with hepatic fibrosis that is the final common pathway of liver injury in most liver diseases [36]. Therefore, accurate quantification for serum A2M level also facilitates to better monitor liver disease progression.

In summary, we profiled proteome alterations between AIH and control samples based on two complementary proteomics methods including 2-DE and iTRAQ. Totally 9 altered proteins were identified in AIH mouse sera by 2-DE MS/MS, furthermore the C3 and A2M were validated to increase in mice model and AIH patient’s sera via iTRAQ and ELISA analysis. Further studies in large samples are needed to demonstrate the specificity, sensitivity and mechanism of C3 and A2M in the diagnosis and pathogenesis of AIH.

## Materials and methods

### Mouse model with autoimmune hepatitis

The following animal experiments were approved by the Institutional Animal Care and Use Committee of Sichuan University, which was also in compliance with all regulatory guidelines.

Female BALB/C mice, 6–8 weeks old, were purchased in the West China Medical Center of Sichuan University (Chengdu, China) to establish AIH models. 24 female BALB/C mice were equally classified into four groups randomly and labeled AIH groups respectively with ConA treatment for 20 h, 30 h and 40 h, and a control group was injected with physiological saline. The mice of AIH groups were injected intravenously via caudal vein with 15 mg/kg ConA (Sigma, USA). Mouse serum was collected from caudal vein after ConA treatment for 20 h to determine ALT level using an automated enzyme assay (Roche Diagnostics GmbH, Mannhein, Germany). And the liver tissues were harvested for histological examination. The experiment of mouse models with AIH was performed 3 repeats.

### AIH patient’s serum

The diagnosis of AIH was made at west China Hospital of Sichuan University according to the criteria defined by the International AIH Group. The Committee on Medical Ethics of West China Hospital approved the following procedures. The venous sera from 34 AIH patients, including 18 males and 16 females (Additional file 1: Table S1), without any drug treatment, were collected from West China Hospital, Sichuan University (Chengdu, P. R. China) with the offers’ informed consent. Normal control sera were obtained from 19 healthy donors (9 males and 10 females) whose gender were matched with the AIH subjects. In addition, in order to compare protein level in other forms of hepatitis, 10 human serum samples were respectively collected from patients with hepatitis B and hepatitis C.

All serum samples were collected and processed according to standard operating procedures to minimize pre-analytical variation [37]. About 3 ml of venous blood was collected into vacutainer tubes (vacutainer plus blood collection tube; Becton Dickinson), allowed to clot for 1 h at 37°C and centrifuged at 2000 g 4°C for 10 min. Serum was then frozen immediately at −80°C.The concentrations of the pooled sera were determined with the Bradford method using bovine serum albumin as a standard.

### Depletion of high-abundance serum proteins

Sera (except hemolysis) were collected from the mouse model or patients with AIH and clotted for 2 h at room temperature. The clotted material was removed by centrifugation at 3000 rpm for 15 min.

In order to enhance the resolution, the main high-abundant proteins were removed from serum using the ProteoExtract™ Albumin Removal column (ProteoExtract™ Albumin Removal Kit; Calbiochem, San Diego, CA). 40 μl of sera was processed with one removal column for each time, and the depletion procedures were performed at room temperature according to manufacturer’s instructions. The collected samples were further concentrated by precipitation using 4 × volume of ice-cold acetone.

### 2-DE

The 2-DE was performed as described previously [18]. One hundred micrograms of the mice serum protein, high-abundance deprived, were suspended in lysis buffer (7 M urea, 4% CHAPS, 60 mM DTT, 2% BioLyte 4–7 (BioRad)) for the isoelectric focusing (IEF) analysis on a 17 cm long IPG (immobilized pH gradient) strips (pH4-7) (Bio-Rad). After IPG strips were rehydrated at 50 V for 12 h, IEF was performed at 20°C with a constant power (50 μA/ IPG-strip) at 250 V for 30 min, linear; 1000 V for 1 h, rapid; linear ramping to 10000 V for 5 h; and finally 10000 V for 6 h. Before the second dimension, the IPG strips were equilibrated in solution A (50 mM Tris–HCl (pH 8.8), 6 M urea, 20% glycerol, 2% SDS and 10 mM DTT) for 15 min, washed with solution B (50 mM Tris–HCl (pH 8.8), 6 M urea, 20% glycerol, 2% SDS, and 200 mM iodoacetamide) for another 15 min. The IPG strip was transferred onto 12% SDS-PAGE gel to separate for second-dimension.

Gels were fixed in 45% ethanol and 5% acetic acid for 2 h, washed with water for 20 min twice, and then incubated in the sensitization solution containing 0.02% sodium thiosulfate for 2 min. After washing with water for 5 min for 3 times, gels were stained in a solution containing 0.1% silver nitrate for 30 min at 4°C. Protein spots were developed in a solution with 2% (w/v) sodium carbonate and 0.04% formaldehyde until spots were clearly visible. Then 1% acetic acid was applied to stop development, finally gels underwent three times of 5-min washes in water.

### Image analysis

Gel images were acquired with Bio-Rad GS-800 scanner under white lights (400–750 nm). Image analysis of the protein spots was performed using the PDQuest software 8.0.1 (Bio-Rad). Detection, normalization and matching of protein spots were automatically performed and manually confirmed. For statistical analysis, student’s t-test (p < 0.05) was performed to compare data from the three repeated experiments. Only such spots that showed consistent and significant differences (over 1.5-fold, p < 0.05) were selected for following MS identification.

### In-gel digestion and MS/MS identification

In-gel digestion of proteins was carried out by using MS-grade trypsin (Promega). Excised spots were cut to about 1-2 mm diameter by using a disposable scalpel and destained by using 50% acetonitrile (ACN) / 50% 50 mM ammonium bicarbonate solution, followed by dehydration in 100% ACN for 15 min. Then each sample was incubated with 10 ng/μL MS-grade trypsin solution overnight at 37°C. Tryptic digests were extracted, and concentrated in a vacuum concentrator at room temperature for MS analysis [38].

As for ESI-Q-TOF MS analysis, the automatic scan rate was 1.0 s with an interscan delay of 0.02 s, and the system was operated at 3.0 kV. Spectra were accumulated until a satisfactory signal/noise (S/N) ratio had been obtained. Parent mass peaks with m/z from 400 to 1600 were selected for MS/MS analysis. The collision energy was chosen to vary between 18 and 57 eV depending on the mass of the precursor.

Data acquisition was performed with MassLynx V4.1 (Micromass) software with automated data processing by the proteinlynx V2.25 software provided with the Waters Corporation. The PKL files were analyzed by the MASCOT search engine (http://www.matrixscience.com). The search parameters were defined as follows: Database, Swiss-Prot 57.2 (466739 sequences; 165389953 residues); taxonomy, mus musculus; enzyme, trypsin; and allowance of one missed cleavage. Carbamidomethylation was selected as a fixed modification and oxidation of methionine was allowed to be variable. The fragment mass tolerance was set at ± 0.3 Da, respectively. Only proteins with at least one peptide exceeding their score threshold (p < 0.05), and with the molecular weight and pI consistent with the gel regions from which the spots were excised, were considered to be positively identified.

### Protein isobaric labeling with iTRAQ reagents

The iTRAQ labeling was performed according to the manufacturer’s protocol (Applied Biosystems) after desalting by acetone precipitation and depletion of abundant proteins. Each type of iTRAQ labeling was performed with 100 μg serum proteins, which were mixed from sera of 3 AIH patients or 3 healthy samples (gender matched). The iTRAQ experiment was performed on two groups for two times. One group of AIH patients included 1 male and 2 females, and another group contained 2 males and 1 female. In each time, the pooled samples were divided into two aliquots (AIH and control group). For blocking cysteine residues, each sample was added with reagents as sequence: 25 μL sample buffer (1 M triethylammonium bicarbonate, TEAB); 1 μL of the denaturant (2% SDS); 2 μL reducing reagent (50 mM tris-(2-carboxyethyl) phosphine, TCEP). The samples were incubated at 60°C for 1 h, and then 1 μL of a freshly prepared 84 mM iodoacetamide solution was added to treat in the dark at room temperature for 30 min.

The proteins were then digested overnight at 37°C with 10 μl of 1 μg/μl trypsin solution (Trypsin Gold, Promega, Madison). And they were labeled with one isobaric amine-reactive tag as following, control sample: iTRAQ Reagent 114; AIH: iTRAQ Reagent 116. The labeled peptides were then mixed and dried. Finally, the pooled iTRAQ-labeled peptide samples were desalted prior to strong cation exchange (SCX)-chromatographic fractionation and LC-MS/MS analysis using a Q-TOF mass spectrometer (Micromass, Manchester, UK), fitted with an ESI or MALDI source (Micromass) coupled with LC Packings Ultimate microcapillary LC system (Dionex, Sunnyvale, CA, USA) [39].

### Database search and iTRAQ quantification

The protein identification and quantification by iTRAQ were achieved using MassLynx Module 4.1 software (Micromass) and MASCOT search engine. The MASCOT searches were run by the following parameters: database, NCBInr 20111206 (16,392,747 sequences; 5,641,810,382 residues); taxonomy, Homo sapiens; enzyme, trypsin; fixed modifications, carbamidomethyl(C), iTRAQ 4plex (N-term), iTRAQ 4plex (K); variable modifications, Oxidation (M), iTRAQ4plex (Y); MS/MS tolerance as 0.3 Da; Instrument type, ESI-QUAD-TOF. Results were scored by using the probability-based score (the ions score is 10 × log (P), where P is the probability that the observed match is a random event), and protein scores are derived from ions scores as a non-probabilistic basis for ranking protein hits.

Protein expression quantification was performed based on iTRAQ data from MS/MS scans, which was displayed as the ratio of peak areas between m/z 114 and 116 Da. The ratios from raw data sets were used to calculate fold changes between control and AIH samples.

The MS raw data for peptide quantification were processed and analyzed by the MassLynx Module 4.1 software. The results were then exported into Excel for manual data interpretation. Generally, most of the protein ratios in the quantification data are close to 1.0 in iTRAQ analysis,the protein ratio lies between 0.785 and 1.275 for almost 95% of the proteins, which indicates that ratios outside these limits are likely to be due to biological, rather than technical variation in a test *versus* control experiment [40]. Therefore, in order to get more accurate data, proteins were usually considered up-regulated or down-regulated when their ratios were >1.3 or <0.7 [41,42]. The error factor (EF) and the 95% confidence interval (95% CI) were calculated for each protein. We set ratios over 1.3 or below 0.7 as statistically significant changes in iTRAQ analysis, with EF < 2 and p <0.05. The median ratio was calculated for each labeling.

### Serum C3 and A2M quantification by ELISA

In ELISA analysis, serum samples were collected from AIH mice at sequential time points after ConA treatment for 20-40 h. The levels of C3 and A2M in serum were quantified using an ELISA assay (Human/Mouse C3 ELISA kit (E90861Hu/E90861Mu); Human/Mouse A2M ELISA kit (E91017Hu/E91017 Mu), USCNK Life Sciences Inc., Wuhan, China) according to the manufacturer’s instructions.

Similarly, serum C3 and A2M levels were also verified in 34 AIH patients, 10 patients with hepatitis B or hepatitis C, and 19 healthy donors by ELISA. The procedures on human serum were fully approved by the Institutional Review Board of West China Hospital.

The data were reported as means ± SD. Statistical significance was assessed by unpaired, two-tailed Student’s t-test when comparing two groups. p < 0.05 was considered statistically significant.

### Biological network analysis with the STRING software

Biological network analysis of C3 and A2M was performed using a web-based software STRING (http://string-db.org). This software STRING aims to analyze data on protein-protein interactions. We queried the interactive network of C3 and A2M with a medium confidence and no more than 10 interactors. The resulting network was presented with a summary of the predicted functional links for the protein, ranked by estimated confidence.

## Abbreviations

ACN: Acetonitrile; AIH: Autoimmune hepatitis; ALT: Alanine transarninase; A2M: Alpha-2-macroglobulin; ConA: Concanavalin A; C3: Complement C3; HAS: Human serum albumin; HSPs: Heat shock proteins; iTRAQ: Isobaric tags for relative and absolute quantification; IEF: Isoelectric focusing; IPG: Immobilized pH gradient; MS: Mass spectrum; SCX: Strong cation exchange; 2-DE: Two-dimensional polyacrylamide gel electrophoresis.

## Competing interests

The authors declare that they have no competing interests.

## Authors’ contributions

Li H and Li G performed experiments and wrote the paper draft; Wu Y and Zhao X collected and validated serum samples; Ma W, Liu Y and Gong F analyzed data. Liang S conceived, instructed the experiments and revised the paper. All authors read and approved the final manuscript.

## Supplementary Material

Additional file 1: Table S1 The serum information from AIH patients in ELISA examination. Click here for file
