# Supplementary material for: Complementary serum proteomic analysis of autoimmune hepatitis in mice and patients
Source: J Transl Med. 2013 Jun 13;11:146. doi: 10.1186/1479-5876-11-146 (PMC3702393; doi:10.1186/1479-5876-11-146)
Supplement: Additional file 1: Table S1 — The serum information from AIH patients in ELISA examination. [file 1479-5876-11-146-S1.doc]

Table S1. The serum sample information from AIH patients in ELISA validation

|  | | Age (years) ^a^ | | |
| --- | --- | --- | --- | --- |
|  |  | 20-40 | 41-60 | 61-80 |
| Serum source Gender | | Sample cases (n) | | |
| AIH patients | Female | 6 | 5 | 5 |
|  | Male | 7 | 8 | 3 |
| Healthy donors | Female | 3 | 3 | 4 |
|  | Male | 4 | 3 | 2 |

^a^ The AIH patients and healthy persons were classified 3 groups based on ages, including young age with 20-40 years, middle age with 41-60 years and old age with 61-80 years.
